# Characteristics of Latrines in Central Tanzania and Their Relation to Fly Catches

**DOI:** 10.1371/journal.pone.0067951

**Published:** 2013-07-18

**Authors:** Seth Irish, Kristen Aiemjoy, Belen Torondel, Faraji Abdelahi, Jeroen H. J. Ensink

**Affiliations:** 1 Environmental Health Group, Faculty of Infectious Diseases, London School of Hygiene and Tropical Medicine, London, United Kingdom; 2 Department of Epidemiology and Biostatistics, University of California San Francisco, San Francisco, California, United States of America; 3 Ifakara Health Institute, Ifakara, Morogoro District, Tanzania; Johns Hopkins University, United States of America

## Abstract

The disposal of human excreta in latrines is an important step in reducing the transmission of diarrhoeal diseases. However, in latrines, flies can access the latrine contents and serve as a mechanical transmitter of diarrhoeal pathogens. Furthermore, the latrine contents can be used as a breeding site for flies, which may further contribute to disease transmission. Latrines do not all produce flies, and there are some which produce only a few, while others can produce thousands. In order to understand the role of the latrine in determining this productivity, a pilot study was conducted, in which fifty latrines were observed in and around Ifakara, Tanzania. The characteristics of the latrine superstructure, use of the latrine, and chemical characteristics of pit latrine contents were compared to the numbers of flies collected in an exit trap placed over the drop hole in the latrine. Absence of a roof was found to have a significant positive association (t=3.17, p=0.003) with the total number of flies collected, and temporary superstructures, particularly as opposed to brick superstructures (z=4.26, p<0.001), and increased total solids in pit latrines (z=2.57, p=0.01) were significantly associated with increased numbers of blowflies leaving the latrine. The number of larvae per gram was significantly associated with the village from which samples were taken, with the largest difference between two villages outside Ifakara (z=2.12, p=0.03). The effect of latrine superstructure (roof, walls) on fly production may indicate that improvements in latrine construction could result in decreases in fly populations in areas where they transmit diarrhoeal pathogens.

## Introduction

Globally, diarrhoea is the leading cause of mortality among children under 5 [1]. Diarrhoeal disease has multiple transmission routes, and its control often requires multiple interventions, which can include improvements in access to water and sanitation, better food and hand hygiene, and drinking water treatment. Synanthropic flies may also play an important role in the transmission of diarrhoeal disease and randomized controlled trials on fly control have shown a reduction in diarrhoea ranging from 22% to 26% [2,3]. A prospective crossover study in Israeli military bases showed an even stronger effect of fly trapping, which resulted in an 85% reduction in shigellosis cases [4].

Pit latrines if designed and managed well can play an important role in diarrhoeal disease control [5]. However, latrines can also provide a feeding and breeding ground for flies. Flies transmit enteric pathogens by landing on, or consuming faecal waste, and then transport this waste on body parts, regurgitate, or defecate on human food and fomites, and so complete the diarrhoea transmission cycle [6]. Despite the widespread availability of latrines and fairly standard contents, not all latrines produce equal numbers of flies [7,8]. Little is known about the reasons for these differences. The pilot study here presented aimed to investigate the association between latrine design, management, the chemical and physical characteristics of pit contents and fly presence in latrines.

## Materials and Methods

### Study Area

The study was conducted as part of a larger study investigating biodegradarion in pit latrines in the Morogoro region of southern-central Tanzania. In rural areas of mainland Tanzania, 71% of households use unimproved toilet facilities, usually an unimproved pit latrine [9]. The study was conducted in pit latrines in the town of Ifakara (08.807’23′′S, 36.840’59′′E) and the villages of Signali and Sululu, roughly 10 km to the north of Ifakara. There are two rainy seasons in central Tanzania, the first (long rains) is from March to May and the second (short rains) is between October and December [10].

### Latrine characteristics

Fifty latrines representing different design and management practices were purposively selected after consultation with village leaders and homeowners in order to monitor latrine fill-up and biodegradation. Householders responded to questionnaires about their latrines and other characteristics were recorded after inspection of the latrines. Characteristics of the latrines recorded included: the age, type of pit-lining (presence and type), floor type, wall type, roof (presence and type), type of latrine use (family or communal, and number of people using the latrine), usage of decomposing additives (usage and type), water table level (flooding), and depth of a latrine (from drop hole to the surface of the pit latrine contents).

### Chemical analysis

Approximately 150 ml of material was collected from the top layer (0-15 cm) of each pit latrine between October 2011 and January 2012. Depending on the consistency of the top material; samples were collected with a standard soil auger (Eijkelkamp, Giesbeek, the Netherlands), or with a sterile 150 ml plastic container attached to the soil auger. *In-situ* temperature and pH measurements were taken with a hand-held meter (HI991003, Hanna Instruments, USA). Pit latrine samples were collected in sterile bags and transported in a cool box for further analysis. Samples were analysed the same day for: total chemical oxygen demand (COD), ammonium, total phosphate, volatile fatty acids (VFA), carbohydrates, protein, moisture content, total and volatile solids.

Samples were homogenised using a Homogeniser pack (Powergen 500, Fisher, UK) following which 1 gram was diluted in 20 ml of _dd_H_2_O. After homogenation and dillution the mixture was passed through a 0.45 µm filter. Samples were analysed using HACH-Lange test kits and methods [11], for total and soluble COD using the dichromate method, for ammonium using the Indophenol blue method, for VFA an esterification method, and for total phosphate a phosphormolybdenum blue method, by using a heatblock (LT20, HACH-Lange, Loveland, USA) and spectrophotometer (DR2800, HACH-Lange, Loveland). Moisture, total and volatile solids content of the samples were measured using standard waste analysis protocols [12], with samples dried at 103-105 °C for total solids, and ignited at 550 °C for volatile solids. Total proteins in each sample was measured using the Lowry assay method [13], while carbohydrate content was assessed by the phenol-sulphuric acid technique [14].

### Exit trap collections

Adult flies were collected using 24-hour exit traps that were placed over the latrine drop-hole. The traps were constructed out of 20-litre plastic buckets with tight fitting lids, and were an adaption of a previously described trap [7]. The bucket was 34.5 cm tall, with a bottom diameter of 28 cm, and a top diameter of 31 cm. A 24 cm diameter hole was cut out of the bucket lid, and covered with fine green 1 mm x 1 mm mesh (non-insecticide treated). The bottom of the bucket was cut out and replaced with a white plastic mesh cone (the mesh was not treated with insecticide and was composed of 3x2 mm squares). The cone was 31 cm tall with a bottom diameter of 25 cm and a top diameter of 7 mm. The cone and lid mesh were attached to the bucket with metal construction staples.

**Figure 1:**
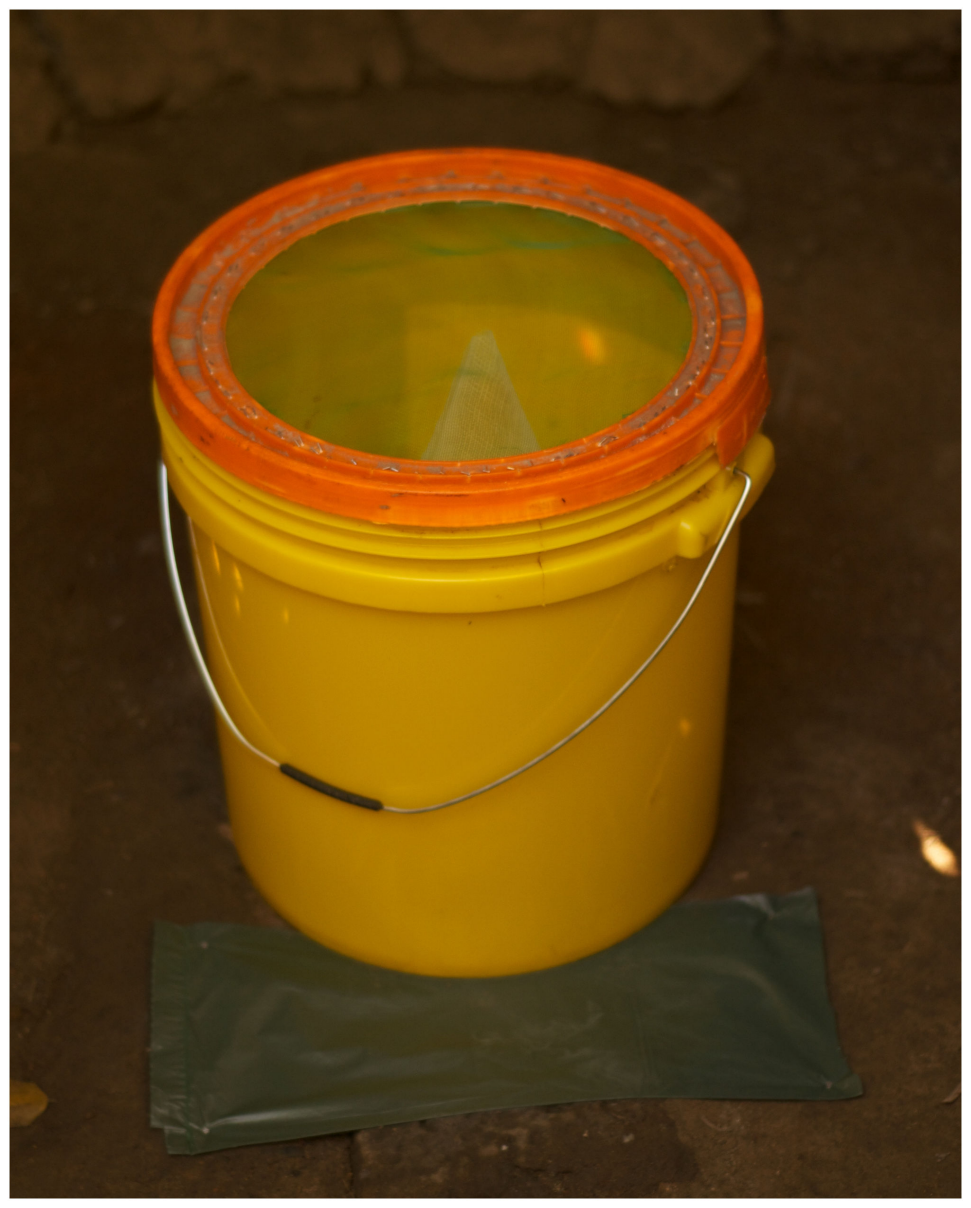
**Drop-hole modification and trap placement.**

Black plastic construction tape and nails were used to adapt the drop-hole and cover other potential exit points (Figure 1). Households were instructed on how to remove and replace the trap when they need to use the latrine. After 24 hours the traps were collected. The traps were transported back to the laboratory and frozen in a -20 °C freezer for 45 minutes to kill the flies. Flies were identified to the family level [15]. All specimens were preserved in ethanol (70% dilution). Each latrine suitable for fly trapping (n=42) was trapped once between July 7 and August 3, 2011.

### Larvae collection

Larvae were sampled from pits by dipping a 100 ml ladle with a 1.3 m handle into the solid contents of the pit before setting the exit trap. One dip was taken from each latrine and was put into a plastic bag for transportation back to the laboratory. In the laboratory, each sample was weighed and all larvae were removed from the sample to be counted and weighed.

### Data analysis

Data was entered into Excel spreadsheets, and analyzed using the Stata 11.2 (Statacorp, College Station, Texas, USA). Latrine characteristics (see Table 1), including the village sampled and chemical characteristics of pit latrine contents, were investigated for associations with the outcome variables (total fly count, Calliphoridae count, or number of larvae per gram) by tabulation. The Calliphoridae family (blowflies) was analyzed as an outcome variable because certain members of this family, particularly *Chryosmyaputoria* , may be of medical importance [8]. The fly counts were analysed for normality using the Shapiro-Wilk test. Total fly counts were log (x+1) transformed before analysis using a linear regression model. The blowfly and larva data were not normalised using a log (x+1) transformation, so a negative binomial regression model was used. Variables were tested for association with the outcome variables (total fly or Calliphoridae counts) in univariable tests, and those significant at the 10% level (p<0.1) in Wald tests were retained for multivariable analysis. In the multivariable analysis, non-significant variables (p<0.05) were dropped one by one in a backwards stepwise analysis until all variables were significant.

**Table 1:** Characteristics of latrine superstructure and use of 42 latrines sampled in and around Ifakara, Tanzania, July–August 2011.

**Characteristic**	**Number of latrines**	**Percentage**
**Roof**		
No	9	31%
Yes	33	79%
**Roof Material**		
Grass/palm leaves	22	67%
Iron sheets	11	33%
**Wall Material**		
Temporary (grass/palm leaves/plastic sheets)	12	29%
Semi-permanent (mud+bamboo)	8	19%
Permanent (bricks)	22	52%
**Floor material**		
Concrete	15	36%
Wood/soil	27	64%
**Pit of latrine**		
Unlined	31	74%
Lined with bricks	11	26%
**Depth of latrine**		
< 1 meter	30	71%
≥ 1 meter	12	29%
**Intensity of use**		
Low < 6 users	14	33%
Medium ≥6 & < 15 users	25	60%
High ≥ 15 users	3	7%
**Age of a latrine**		
< 1 year	17	40%
≥1 & < 5 years	13	31%
≥ 5 years	12	29%
**Use of additive**		
No		
Yes	23	55%
**Type of additive used**	19	45%
Bleach/Oil	3	16%
Ash	16	84%
**Groundwater floods latrine**		
No	13	31%
Yes	29	69%
**Water stored in latrine**		
No	6	14%
Yes	36	86%

### Ethics

The Ifakara Health Institute’s (IHI) review board, National Institute for Medical Research (NIMR) in Tanzania, and the LSHTM granted ethical approval for this study (IHI 14-2-10, NIMR 1143, LSHTM 5659). Community meetings were held to introduce the study, and all study participants provided written informed consent.

## Results

### Latrine characteristics

The characteristics of the latrines are displayed in Table 1. The majority of latrines did not have a lined pit or a concrete slab. The mean depth of the latrine from the drop-hole to the pit contents was 1.48 m (95% CI: 1.26-1.65 m), with a minimum depth of 0.46 m and a maximum depth of 2.8 m. The majority of latrines were used by a single household, with a mean number of users of 8 (ranging from 2 to 35). The mean age of latrines was 4 years (95% CI: 2.5-5.4 years).

### Chemical characteristics

The chemical analysis of the samples collected from the top of a pit latrine showed a wide range of conditions (Table 2). The top layer in some latrines was primarily liquid, while in others it was more solid. The amount of organic matter available also ranged widely between latrines.

**Table 2:** Characteristics of latrines contents sampled for flies (n=42), reported as dry weight.

**Parameter**	**Unit**	**Mean**	**Min – Max**
COD total	g/kg	728.3	45.1–1971.4
Moisture content	%	70.0	0.0-72.7
NH_4_	g/kg	8.3	0.1–38.4
Ph		7.0	5.3–8.2
Temperature	°C	28.6	25.5–33.0
Total Phosphate	g/kg	12.3	1.0–87.9
VFA	g/kg	67.1	0.6–576.8
Protein	%	27.2	0.9–67.4
Carbohydrate	%	75.3	0 - 100
Total solids	%	30.0	0-72.7
Volatile solids	%	49.8	0-91.0

### Exit trap collections

Of the 50 latrines examined, only 42 were suitable for fly trapping. Three had collapsed, three had flush/septic tank systems, and two were public latrines with a high number of users, making a 24-hour exit trap collection impossible. A total of 5,223 flies were collected in the 42 trap collections of the study, ranging from 0 to 1542 per trap. The most common family caught was Psychodidae (48% of all caught), followed by Culicidae (32%), Calliphoridae (19%), Syrphidae (0.3%), Stratiomyidae (0.2%), Sarcophagidae (0.03%) and unidentified flies (0.4%) (Table 3). Although flies were not identified to species, *Chrysomya putoria* (Calliphoridae), *Hermetiaillucens* (Stratiomyidae), and *Culex* *quinquefasciatus* (Culicidae) have been identified from exit traps on latrines in and around Ifakara (Seth Irish, unpublished data).

**Table 3:** Families of flies (Diptera) collected in latrine exit-traps.

**Family of Fly**	**Total**	**Mean catch per latrine (standard deviation)**	**Range**
Calliphoridae	1004	23.9 (54.1)	0-324
Psychodidae	2488	59.2 (123.9)	0-625
Culicidae	1684	40.1 (232.1)	0-1502
Sarcophagidae	2	0.05 (0.2)	0-1
Stratiomyidae	8	0.2 (0.5)	0-2
Syrphidae	18	0.4 (1.3)	0-8
Unknown	19	0.5 (1.0)	0-4
Total	5223	124.4 (281.9)	0-1542

The absence of a roof was found to have a significant association (p=0.003) with log-transformed total adult fly numbers. In latrines with roofs, a geometric mean (x+1) of 14.6 flies (95% CI: 7.6-27.8) were caught as compared to 121.2 flies (95% CI: 35.5-414.0) in latrines without roofs. The results by family are given in Table 4.

**Table 4:** Geometric means (95% confidence intervals) for families of flies when roof is absent and present.

**Family of Fly**	**Roof absent (n=9)**	**Roof present (n=33)**
Calliphoridae	24.5 (5.0-121.0)	4.2 (2.5-6.8)
Psychodidae	60.0 (14.2-254.3)	4.6 (2.4-8.5)
Culicidae	1.5 (0.9-2.5)	2.0 (1.2-3.6)
Sarcophagidae	1.1 (0.9-1.3)	1.0 (1.0-1.1)
Stratiomyidae	1.4 (1.0-1.8)	1.1 (1.0-1.2)
Syrphidae	1.7 (0.9-3.0)	1.1 (1.0-1.2)
Unknown	1.3 (0.8-2.0)	1.3 (1.1-1.5)
Total	121.2 (35.5-414.0)	14.6 (7.6-27.8)

The numbers of Calliphoridae collected in exit traps had a negative binomial distribution and were analysed using a negative binomial regression model. The variables that significantly affected the number of Calliphoridae collected in exit traps were the material used for the walls of the superstructure of the latrine, the trap used, total solids and volatile solids (Table 5). The highest numbers of Calliphoridae were collected in latrines with temporary superstructures, and the fewest were collected in latrines with permanent superstructures. Fewer Calliphoridae were collected in latrines with low proportions of total solids, while the opposite was true for volatile solids (Table 5).

**Table 5:** Median number and interquartile range of Calliphoridae collected in latrines with different materials used for walls of the latrine superstructure, different total solid levels, and different volatile solid levels.

**Characteristics**	**n**	**Median**	**Interquartile range**
**Materials used for walls**			
Temporary structure (palm leaves/grass/ plastic)	12	23	0.5-88
Semi permanent structure (wattle and daub)	8	10	4-14.5
Permanent structure (bricks)	22	1	0-9
**Total solid range (%)**			
0 - 24.9	21	1	0-7
25-49.9	11	17	2-28
50–74.9	10	15	0-53
**Volatile solid range (%)**			
0 - 24.9	14	15	0-53
25-49.9	3	2	0-21
50–74.9	14	3	0-12
75–99.9	11	0	0-17

In addition to the flies collected in the traps, cockroaches were also collected in 4 of the traps. Forty-one cockroaches were collected, but 36 of these were collected in one trap collection.

### Larval collections

Of the 50 latrines, only 24 latrines were shallow enough (depth < 1.30 m) to dip for larvae. Samples ranged in weight from 27.6 to 147.9 g and numbers of larvae in each sample ranged from 0 to 496. When the latrine characteristics were analysed for association with the number of larvae per gram, only the village (p=0.045) was significantly correlated with numbers of larvae per gram.

## Discussion

Psychodidae were the most commonly collected fly family in this study. Most of these flies were in the Psychodinae subfamily, and appear not to have a role in mechanical transmission of pathogens, though they have occasionally been involved in myiasis [16,17], or a cause of insect allergy [18].

The other two families of Diptera that were collected in high numbers in this study were Calliphoridae and Culicidae. Calliphoridae (particularly *Chrysomya putoria*) and Culicidae (primarily *Culex* *quinquefasciatus* ) have been noted by several studies on African latrine fauna [7,8,19]. *Culex* *quinquefasciatus* is an important vector of *Wuchereria* *bancrofti* on the east coast of Africa, and an important nuisance biting mosquito throughout the world [20]. *Chrysomya putoria* is a putative mechanical vector of diarrhoeal pathogens, though its role in pathogen carriage is not fully understood [7].

Interestingly, no Muscidae were collected in the exit traps. *Musca* *domestica* is one of the most common synanthropic flies [21], and has been implicated as a mechanical vector for several diseases [22–24]. *Musca* *domestica* has been found to breed in high numbers in latrines in some studies in the United States [25,26] but not in others [27]. In several studies on African latrines, as in the present study, *Musca* *domestica* does not seem to be a primary species collected in exit traps over drop holes [7,8,28,29].

This study is a cross-sectional pilot study which took place towards the beginning of the long dry season (July/August). Insect fauna in latrines can vary by season [27], so further collection would be needed to evaluate any seasonal effects.

Only one variable (presence/absence of a roof over the latrine) was found to have a significant association with total fly numbers. This difference seems to have been primarily influenced by the numbers of Calliphoridae and Psychodidae. The absence of a roof may have provided easier access to flies to enter the latrine. Early work on the development of the ventilated improved pit latrine in Zimbabwe also found lower number of flies in latrines with a roof [30]. The absence of a roof may result in a similarly open-access latrine. Another factor to consider is the amount of light that is entering a latrine, which may also affect flies entering and exiting through the drophole, which was shown by work in Zimbabwe [30]. Light entering the latrine might also result in important differences of temperature, which could also affect the attractiveness of the latrine to flies, as well as having an effect on the upper layers of the pit latrine contents.

When only Calliphoridae were considered, four variables were significantly associated with the trap counts. Two of these variables were positively associated with increased Calliphoridae catches, the latrine superstructure and total solids in pit latrine contents. The material used for construction of the walls of the superstructure may be an indicator of access to the flies to enter the latrine, as the most solidly built superstructures had the lowest median number of flies and latrines with temporary superstructures had more blowflies. Increased total solids in pit latrine contents were also associated with increased numbers of flies. The individual trap used to collect flies was another variable that was significantly associated with Calliphoridae catches. The traps were constructed of the same type of materials and were prepared in the same way, however, they were different colours. The colour of the lid of a fly trap was not shown to significantly affect the number of *Chrysomya putoria* collected in traps in The Gambia, however, the opacity of traps did affect the trap catch [31]. In future studies, the same colour of exit trap should be used to avoid any variation due to this factor. The total and volatile solids measured in the pit latrine contents indicate the amount of all solids and the organic matter of animal or plant origin present in the latrine sample, respectively[12]. . Interestingly, there were more Calliphoridae collected in traps where the volatile solids were lower. Volatile solids have been associated with greater attraction for flies [32], but it may be that the levels of volatile solids present in the latrines were affected by the presence of larvae or other factors. Furthermore, recent work on the chemical composition of pit latrine contents shows that these contents are far from homogeneous (Ensink et al., in preparation) and the samples were not collected at the same time as the fly trapping, so further work is needed before the relationship between flies and pit latrine contents can be completely understood.

The number of larvae per gram of the pit latrine contents was only significantly correlated with the village where the latrine was located. This may indicate that other aspects of the environment, such as the physical properties of soil or diet of the local population, can also have an effect on the production of flies from latrines. However, differences between villages were not found for adult flies and only one sample was taken per latrine, so further research is needed to understand the reason for this effect.

Of the significant associations found in this study, the most interesting appear to be the relation of superstructure to the fly counts. Both the presence of roof (for total fly catch) and wall structure (for Calliphoridae) were significantly associated with fly catches. This is not a surprising finding, particularly as the ventilated improved pit latrine has been designed with the idea of preventing fly breeding and this latrine has solid walls, no windows, and a roof. However, in areas where building of this type of latrine is not conducted, construction of latrines with roofs and solid walls may result in reduced populations of flies visiting or breeding in latrines.

Public health officials and policy makers that promote sanitation often have a very different agenda from those that use sanitation. Public health officials promote sanitation based on public health reasoning, assuming that simply providing facilities will mean that they will be used by the recipients. The main reasons why most people want sanitation often have less to do with health, than with dignity, privacy, status and comfort [33]. The promotion of sanitation involves understanding why people want sanitation and offering them suitable options [34]. Latrines that promote fly-breeding, even those that might not directly be linked with disease transmission, still pose a public health risk, as they might deter people from using facilities, and therefore revert back to open defeacation.

## Conclusions

In this study, the most common flies collected were moth flies (Psychodidae), mosquitoes (Culicidae), and blow flies (Calliphoridae). The total numbers of flies collected from latrines were positively associated with latrines without roofs. The numbers of blowflies were positvely associated with the type of superstructure and the total solids in pit latrine contents. Other factors affecting fly catch included the volatile solids in pit latrine contents, and the location of the latrine. All of these variables might be manipulated for better control of flies and should be considered when planning sanitation for low-income communities.
